# The effect of different acidic irrigation solutions on the pushout bond strength of root canal filling

**DOI:** 10.34172/joddd.2023.36920

**Published:** 2023-04-03

**Authors:** Funda Fundaoğlu Küçükekenci

**Affiliations:** Department of Endodontics, Faculty of Dentistry, Ordu University, Ordu, Turkey

**Keywords:** Bioceramics sealer, Bond strength, Irrigation solution, Resin sealers

## Abstract

**Background.:**

This study investigated the effects of different acidic solutions used as the final irrigation on the push-out bond strength (PBS) of resin-based and bioceramic-based root canal sealers.

**Methods.:**

100 single root and canal human incisors were selected and decorated. Root canal shaping was done with ProTaper Next rotary files up to X4 and rinsed with 5 mL of 5.25% NaOCl between each file. Then, teeth were divided into five main groups according to the final irrigation (n=20). Group 1: glycolic acid; Group 2: phosphoric acid; Group 3: citric acid; Group 4: EDTA and group 5: saline. Then, each group was divided into two subgroups according to the canal sealer used (n=10). The groups filled with bioceramic-based sealer (bioserra) were named A, and the groups filled with resin-based sealer (AH Plus) were called B. PBS test was applied to one of the two samples obtained from the coronal third of each root. The data were statistically analyzed using a two-way analysis of variance and Tukey’s HSD test (α=0.05).

**Results.:**

Statistically, the highest PBS value was obtained in group 2A (4.81±0.03 MPa), which was irrigated with phosphoric acid and filled with bioserra, and the lowest PBS value was obtained in group 5B (1.10±0,03), which was irrigated with saline and filled with AH Plus (*P*<0.05). There was a statistical difference between all groups except group 1A and group 3A (*P*<0.05).

**Conclusion.:**

The bioceramic-based root canal sealer (bioserra) bond strength is superior to resin-based (AH Plus). Phosphoric acid, glycolic acid, and citric acid can be an alternative to EDTA.

## Introduction

Root canal treatment aims to shape the root canals chemomechanical followed by a three-dimensional sealed canal filling.^1^ Due to difficulties in root canal anatomy, it is known that untouched dentin surfaces remain in a mechanically shaped root.^2^ Microorganisms and infected dentin surfaces in these areas that cannot be cleaned mechanically adversely affect the achievement of root canal therapy.^3^ Therefore, chemical irrigation and disinfection are required to remove the smear layer formed during mechanical shaping and disinfection of these untouched areas.^4^ Various irrigation solutions are used for this purpose.^5^ However, a single irrigation solution cannot achieve complete success. Therefore, using more than one solution together and in a particular order is recommended.^6^ Sodium hypochlorite (NaOCl) is often preferred as an irrigation solution due to its high antimicrobial activity and organic tissue dissolving effect.^7^ In addition, it is recommended to use acidic solutions that cause chelation or decalcification after NaOCl dissolves organic tissues by proteolytic activation.^8-10^ Although the combination of NaOCl and ethylenediamine tetraacetic acid (EDTA) is seen most frequently in clinics, it has recently been reported that acidic solutions such as peracetic acid, glycolic acid, citric acid, phosphoric acid can be used for this purpose.^11^ EDTA forms soluble calcium chelates by reacting with the calcium ions in the dentin structure. This decalcifying feature can be used as a solvent for the inorganic component of the smear layer. However, it has little or no effect on organic tissues. Therefore, it is used together with NaOCl.^12^ Prado et al^13^ reported that citric acid and phosphoric acid could achieve similar results to EDTA. Also, the ability of glycolic acid to remove the smear layer enables it to be used as a final irrigation solution.^14^ Elimination of the smear layer is significant as it allows the bond of sealers to the dentinal tubules. Removing the smear layer may prevent the root canal sealer from mechanically locking to the root dentin and displacing the canal filling material.^15^ Although gutta-percha is the mainly used canal obturation material, it could not adhere to dentine alone.^16^ There are various canal sealers, such as glass ionomer-based, resin-based, calcium silicate-based, and silicone-based root canal sealers, that could be used for gutta-percha to adhere to dentin. The canal sealers based on epoxy resin-based are frequently used due to their excellent physical properties, dimensional stability, low solubility, and micromechanical bonding to dentin.^1^ Calcium silica-based canal sealers are called bioceramics because they form a bioactive surface that allows hard tissue to be built. During the hardening reaction of bioceramics, a surface containing calcium hydroxide, calcium silica hydrate, and calcium phosphate, called the mineral infiltration zone, is formed. This surface provides apatite nucleation and allows the root canal filling material to be attached to the dentin with tags.^17^ The previous studies showed that bioceramics sealers’ bond strength is superior to conventional root canal sealers.^3,18^ However, the efficacy of the present or absent smear layer on the bond strength of bioceramic pastes is controversial. Shokouhinejad et al^19^ reported that the bond strength of bioceramics is not affected by the smear layer. Studies examining the effect of different irrigation protocols aimed at removing the smear layer on bonding calcium silica-based pastes to dentin are limited. The present study aimed to investigate the effect of final irrigation solutions on the bond strength of bioceramic and resin sealers. The null hypothesis is that the irrigants used in the present study will not affect the bond strength of the sealers used.

## Methods

 In this study, the sample size was calculated based on a power analysis using G*Power software version 3.1 (Universität, Düsseldorf, Germany) at an alpha error probability of 0.05 and a power of 95% (effect size = 0.85). The power analysis showed that a total of 100 samples were required for groups. A single root and canal human incisor that was extracted for orthodontic, periodontal, or prosthetic reasons was used (N = 100). Teeth were kept in distilled water until use. Radiographs were taken from each tooth in the mesiodistal and buccolingual direction and evaluated for internal or external resorption, previous root canal treatment, and additional canals. Teeth with additional canals, resorption, or previous endodontic treatment were excluded. The included teeth were separated from their crowns with diamond separators, and the root lengths were standardized to be 12 mm. The length 1 mm shorter than the apical exit of the file was determined as the working length. Root canal preparation was performed with the ProTaper Next file system up to X4 file (Dentsply, Ballaigues, Switzerland). 2.5% NaOCl was used as an irrigation solution between each file. After the preparation, specimens were rinsed with 5 ml of 2.5% NaOCl, followed by 5 ml of distilled water for 60 seconds. Appropriate paper points were used to dry the root canals. The specimens were divided into five main groups with 20 teeth in each group according to the final irrigation solution, and then divided into subgroups A and B according to the canal sealer used (n = 10).

- Group 1: 60 seconds of irrigation 5 mL 17% glycolic acid^14^
- Group 2: 90 seconds of irrigation 5 mL 37% phosphoric acid^13^
- Group 3: 60 seconds of irrigation 5 mL 10% citric acid^13^
- Group 4: 60 seconds of irrigation 5 mL 10% EDTA^2^
- Group 5: 60 seconds of irrigation 5 mL saline solution.

After the irrigation protocol, the roots in groups A were filled with a bioceramic sealer (bioceramic sealer (Bioserra®, Dentac, Turkey) and gutta-percha. The roots in groups B were filled with resin-based root canal sealer (AH Plus; Dentsply, Konstanz, Germany) and gutta-percha. The canal orifices were restored with Orafil G and kept for 48 hours at 100% humidity and 37ºC temperatures for the sealer to set completely. The specimens were embedded in auto-polymerized acrylic resin blocks (Meliodent, Bayer Dental, Leverkusen, Germany) and sectioned into two slices for the third coronal region by using a precision cutting machine (Mecatome T180, Presi Metallography, Eybens, France) with water cooling to obtain 1 mm horizontally sections.

A universal testing instrument (Autograph AGS X; Shimadzu Co, Japan.) was used to conduct the push-out bond strength (PBS) test. One sample was selected from the coronal region for the PBS test (n = 10). PBS test was applied from apical to coronal with a 1 mm diameter plugger at a crosshead speed of 0.5 mm/min until the canal filling was dislocated. The bond failure force recorded in Newtons (N) is the peak force displacing the filling. The N value was converted to megapascals (MPa) for each sample by dividing the N value into the total bonding area (mm^2^). The total bonding area was calculated as π (r_1_ + r_2_) h, where h is the thickness of the sample, r_1_ is the apical radius of the root canal, r_2_ is the coronal radius of the root canal, and π = 3.14.

###  Statistical analysis

Statistical software (IBM SPSS Statistics, v20.0; IBM Corp) was used to analyze the data. According to the Levene homogeneity test, the variables were normally distributed (*P* = 0.939). The PBS results were evaluated with the 2-way analysis of variance (ANOVA) for descriptive statistics and the effects of the irrigation solution, sealer type, and their interactions. The mean of the PBS values was compared using Tukey honestly significant difference (HSD) tests (α = 0.05).

## Results

According to the 2-way ANOVA results, the final irrigation solutions, the type of root canal sealer, and their interaction significantly affected the PBS values (*P* < 0.05) (Table 1). The mean PBS values (MPa), the standard deviations (SD), and the multiple comparisons of PBS values according to the Tukey HSD test are presented in Table 2.

**Table 1:** Two-way ANOVA test results

**Variable (Source)**	**Sum of squares**	**df**	**Mean squares**	**F**	***P***
Sealer type (A)	11.384	1	11.384	12620.705	< 0.000
Final irrigation solution (B)	96.709	4	24.177	26803.936	< 0.000
A × B	502	4	0.125	139.092	< 0.000
Error	0.081	90	0.001	-	-
Total	973.624	100	-	-	-

*P <*0.05 indicates a significant difference.

**Table 2:** Push-out bond strength values (Mean ± SD) in MPa of the experimental groups

**Group**	**Bioserra/gutta-percha (Group A)**	**AH Plus /gutta-percha (Group B)**
Group 1	3.50 ± 0.03^g^	2.92 ± 0.03^e^
Group 2	4.81 ± 0.03^i^	4.00 ± 0.03^h^
Group 3	3.50 ± 0.03^g^	2.63 ± 0.03^d^
Group 4	3.00 ± 0.03^f^	2.37 ± 0.04^c^
Group 5	1.60 ± 0.03^b^	1.10 ± 0.03^a^

SD: Standard deviation. *Tukey HSD comparisons of PBS values (MPa) were presented as superscripts, and significant differences were indicated with different letters (P < 0.05).

The highest PBS value was determined in Group 2A (4.81 ± 0.03 MPa), which was irrigated with phosphoric acid and filled with bioserra®. The lowest PBS value was obtained in Group 5B (1.10 ± 0.03), which was irrigated with saline solution and filled with AH Plus. There was a statistically significant difference between all groups except Group 1A and Group 3A (*P* < 0.05). Regardless of the final irrigation solutions, the PBS values of the bioserra® sealer were obtained statistically higher than the AH plus sealer in each group (*P* < 0.05).

## Discussion

The prognosis of root canal treatment is directly related to the adhesion of the root canal filling to the dentinal walls.^20^ If the filling material adheres adequately to the root walls and maintains its dimensional stability, the dislocation of the canal filling material against both restorative procedures and chewing forces is prevented, thus reducing the risk of re-infection due to the possibility of leakage. Endodontic sealers resist the forces that will dislodge the canal filling by preserving the integrity of the dentin sealer with micromechanical or frictional adhesion.^21^

Different tests, such as micro tensile, shear bond strength, and PBS, can measure the bond strength between the root canal sealer and dentin. However, PBS is used more frequently in the adhesion evaluation because it better mimics the clinical situation, is easy to administer, and interprets the data.^22,23^ Therefore, in the present study, the PBS test was used to examine the adhesion of AH plus and bioserra® canal filling sealer to the root dentin wall. The better the adhesion capability of the root canal sealers is to the dentin, the higher the bond strength between the canal filling and the root dentin wall.^24^ The tubular density in the radicular dentin decreases from the coronal to the apical region, so it was seen as appropriate to evaluate the sealer-dentin bond strength in the coronal region of the root in this study. For adequate chemical or micromechanical bonding, there must be close contact between the adhesive material and the bonded surface, which is related to the wetting ability of the surface.^25^ The wettability of the surface is affected by the surface treatments.^26^ Irrigation solutions used during chemomechanical shaping cause changes in intraarticular dentin’s physical and chemical structure.^27,28^ This dentin structure can affect root canal sealers’ adhesion in different ways.^29^ The effect of irrigation with NaOCl and EDTA on the sealer has been investigated, but no study evaluated other chelation agents that can be used instead of EDTA. The present study assessed the PBS in the final irrigation with glycolic acid, phosphoric acid, citric acid, and EDTA on bioceramic-based bioserra® and resin-based AH plus root canal sealer.

According to the results of the present study, statistically higher PBS was observed in which a chelating agent was used to remove the smear layer, compared to which was not used as a chelation agent in both sealer groups. In the subgroups using a bioceramic-based sealer, PBS was statistically higher than in the subgroups using an epoxy resin-based sealer. So, PBS values were affected by the chelation agent and sealer. Thus, the null hypothesis was rejected. In the previous study, it was reported that the reason for the low PBS detection in the saline groups in which the smear layer was not removed was that the sealer contacted the dentinal tubule more in the groups in which the chelation agent was used.^30^ In the present study, the lowest PBS values were observed in the saline solution groups in both sealer groups, which shows the negative effect of the remaining intact smear layer on the bond strength. In addition, in parallel with the previous studies,^3,18^ it was determined that the PBS values in the groups using bioceramic-based sealants were higher than those using resin-based sealants in the present study. This situation can be attributed to since NaOCl creates a potent inhibition at the sealer-dentin interface by the decomposition of oxygen and sodium chloride and affects the polymerization of the resin.^31^ In addition, this may be due to the hydrophilic nature of bioceramic sealers, which uses moisture from tubular dentin during setting.^32^

According to the results of the present study, the highest bond strength was seen in the experimental groups using after 32% phosphoric acid irrigation both bioserra® sealer (4.81 ± 0.03 MPa) and AH plus sealer (4.00 ± 0.03 MPa) were used in the groups (*P* < 0.05). This result was similar to the previous study, which evaluated the effect of irrigation solutions (EDTA, phosphoric acid, and MTAD) on PBS of bioceramic and resin-based sealer to dentin and determined the highest PBS value in the phosphoric acid group.^28^

The higher PBS values of phosphoric acid were explained by the fact that more dentin tubules were exposed due to dentin demineralization, and the resin matrix formed a stronger bond with this layer.^13,33^ In addition, it was thought that phosphoric acid completely removes the inorganic components of the smear layer and exposes the collagen fibrils of the dentin and that these collagen fibrils and hydrophilic bioceramic-based sealers create dentin hybridization, which provides high bond strength.^34^ However, the effect of such a strong acid on periapical tissues when extrusion into periapical tissues is unknown. This situation should be investigated in further studies. The highest bond strength after phosphoric acid was observed in bioserra® (3.50 ± 0.03 MPa) and AH plus sealer (2.92 ± 0.03 MPa) groups after glycolic acid was used. Demirbaş et al^35^ showed that glycolic acid causes higher PBS than EDTA use. This result can be explained by the fact that glycolic acid has a lower pH than EDTA and a lower molecular weight than EDTA. Bernstein et al^36^ reported that glycolic acid could induce collagen synthesis. The high bond strength in glycolic acid groups may be its positive effects on the collagen matrix.^35^ The bond strength in the group irrigated with citric acid was similar to the irrigated glycolic acid. This situation suggests that the effect of these two acids on the smear layer may be similar. It has been shown in previous studies that the use of EDTA removes the smear layer, exposes the dentinal tubules, and increases the connection of the AH plus sealer with micromechanical retention. In our study, the bond strength was found to be higher in the groups using EDTA compared to the saline groups, but EDTA showed lower bond strength than other chelation agents.^22,37,38^

 One of the limitations of the present study is the use of two-dimensional radiographs for the initial teeth evaluation. These radiographs cannot give precise information about root canal anatomy or the resorption areas in the canal that affect the sealers’ connection to the dentin. Therefore, in future studies, CBCT, which provides a three-dimensional image, can be used both in the evaluation of the initial condition of the canal and in the evaluation of the quality of the canal filling. Radicular dentin has a non-uniform structure that decreases tubular density from coronal to apical. After chemomechanical shaping, the difference in dentin structure becomes more pronounced. Therefore, samples from the coronal third of the root were tested in this study. This situation is another limitation of the present study. Still, since in clinical conditions the connection is on the entire root surface, it is recommended to test other root regions in future studies. The other limitations of the present study are the inability to simulate the chewing forces that teeth are exposed to in the mouth in-vitro conditions and the ability to measure the bond strength of sealers only in the corona-apical direction. Future investigations are required that force is applied in different directions to measure the bond strength. On the other hand, in future studies, the biocompatibility of tested irrigation solutions and the effect of irrigation solution on the bond strength of teeth belonging to different ages, sex, and regions and the different third parts of these teeth should be evaluated.

## Conclusion

 The results of the present study showed that phosphoric acid, glycolic acid, and citric acid can be evaluated as chelation agents that can be used as an alternative to EDTA. Also, bioserra®, a bioceramic-based sealer, has a higher PBS value when used with tested irrigation solutions.

## Acknowledgments

 The author has no financial interest related to this study disclosure.

## Competing Interests

 The author declares that she has no known competing financial interests or personal relationships that could have influenced the work reported in this paper.

## Ethical Approval

 This study was approved by Ordu University Clinical Research Ethics Committee (242/2022).

## Funding

 None.
